# Diagnosis of Chronic Pulmonary Aspergillosis: Clinical, Radiological or Laboratory?

**DOI:** 10.3390/jof9111084

**Published:** 2023-11-06

**Authors:** Aleksandra Barac, Ankica Vujovic, Ana Drazic, Goran Stevanovic, Bianca Paglietti, Katarina Lukic, Maja Stojanovic, Mihailo Stjepanovic

**Affiliations:** 1 Clinic for Infectious and Tropical Diseases, University Clinical Center of Serbia, 11000 Belgrade, Serbia; ankica.vujovic88@gmail.com (A.V.); goran_drste@yahoo.com (G.S.); 2 Faculty of Medicine, University of Belgrade, 11000 Belgrade, Serbia; drazic998@gmail.com (A.D.); dr.maja.stojanovic@gmail.com (M.S.); mihailostjepanovic@gmail.com (M.S.); 3 Department of Biomedical Sciences, University of Sassari, 07100 Sassari, Italy; biancap@uniss.it; 4 Center for Radiology and MRI, University Clinical Center of Serbia, 11000 Belgrade, Serbia; katarina.krstic86@hotmail.com; 5 Clinic of Allergy and Immunology, University Clinical Center of Serbia, 11000 Belgrade, Serbia; 6 Clinic for Pulmonology, University Clinical Center of Serbia, 11000 Belgrade, Serbia

**Keywords:** chronic pulmonary aspergillosis, clinical symptoms, radiological findings, laboratory diagnosis, antibody

## Abstract

Chronic pulmonary aspergillosis (CPA) is a chronic progressive lung disease associated with a poor prognosis and a 5-year mortality rate of approximately 40–50%. The disease is characterized by slowly progressive destruction of the lung parenchyma, in the form of multiple cavities, nodules, infiltrates or fibrosis. CPA can be challenging to diagnose due to its non-specific symptoms and similarities with other respiratory conditions combined with the poor awareness of the medical community about the disease. This can result in delayed treatment even for years and worsening of the patient’s condition. Serological tests certainly play a significant role in diagnosing CPA but cannot be interpreted without radiological confirmation of CPA. Although many data are published on this hot topic, there is yet no single definitive test for diagnosing CPA, and a multidisciplinary approach which involves a combination of clinical picture, radiological findings, microbiological results and exclusion of other mimicking diseases, is essential for the accurate diagnosis of CPA.

## 1. Introduction

Chronic pulmonary aspergillosis (CPA) is a fungal infection caused by the inhalation of spores from *Aspergillus* spp., most commonly *Aspergillus fumigatus*, although similar clinical and radiological presentation can also be attributed to other fungi [1]. The reason and the way of *A. fumigatus* domination is still unclear to clinicians, which is motivating scientists to explore its pathogenic mechanisms in order to make diagnosis and therapy as effective as possible. CPA is a chronic progressive lung disease associated with a poor prognosis [2] and a 5-year mortality rate of approximately 40–50% [3]. It mostly affects individuals who are immunocompetent or mildly immunocompromised due to an underlying lung condition, such as chronic obstructive pulmonary disease (COPD), pulmonary tuberculosis (PTB), nontuberculous mycobacterial infections (NTM), or lung cancer [4]. The disease is characterized by slowly progressive destruction of lung parenchyma, in the form of multiple cavities, nodules, infiltrates or fibrosis [5].

It is difficult to estimate the incidence and prevalence of CPA; however, the global burden of disease is increasingly being recognized [6]. Global epidemiological data show that approximately 3 million people suffer from CPA [7], while it is estimated that almost half of the global CPA burden is related to PTB, with 1.2 million infections worldwide [8] and at least the same number or more cases from other pulmonary disorders including COPD, NTM, sarcoidosis, post-pneumothorax, allergic bronchopulmonary aspergillosis (ABPA) and rheumatoid arthritis [9]. Previously treated PTB is recognized as one of the most important risk factors for CPA [10]. Patients with a history of PTB and residual cavities have the highest risk, with 5–35% of patients developing CPA as a sequel to PTB [8].

**Figure 1:**
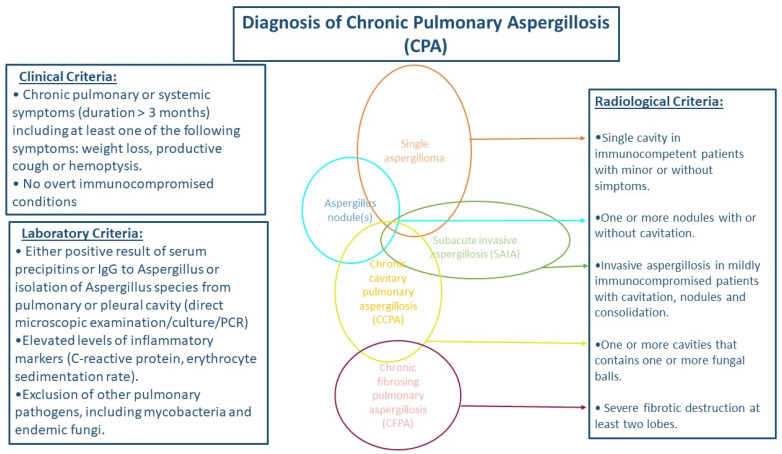
Schema for diagnosis of chronic pulmonary aspergillosis.

Estimation of CPA prevalence in different geographic regions shows high prevalence in Philippines, Pakistan, and Romania (78, 70, 59 per 100,000 respectively). The estimated prevalence of CPA in different regions appears to have increased in the last decade, probably due to the more precise diagnostic tools and guidelines [7]. The diagnostic criteria for CPA have been defined by the European Society for Clinical Microbiology and Infectious Diseases (ESCMID), the European Respiratory Society (ERS) and the European Confederation of Medical Mycology (ECMM) and include: (i) a consistent appearance in thoracic imaging (preferably by CT) for ≥3 months, (ii) direct evidence of *Aspergillus* infection or immunological response to *Aspergillus* spp., and (iii) exclusion of alternative diagnoses [11] (Figure 1).

While CPA diagnosis still presents a challenge, due to the similar presentation with other chronic respiratory diseases, we aimed to review novel data on the laboratory, clinical and radiological diagnosis of CPA. 

## 2. Ethiopathogenesis

As mentioned, *A. fumigatus* is the most common cause of CPA [12]. This is a trimorphic filamentous fungus with vegetative mycelium in nature and in patients. It was long believed that *A. fumigatus* was an exclusively asexually reproducing organism, but it is now accepted that *A. fumigatus* also has the ability to reproduce sexually/parasexually and/or asexually, which is very important for the adaptive potential of *Aspergillus* which can include resistance development in a patients with CPA [13]. Nevertheless, the majority of reproduction in nature occurs asexually, with *A. fumigatus* sporulation profusely to generate small, hydrophobic conidia that can be dispersed aerially across significant distances [14]. In recent decades, *A. fumigatus* has shown a multifactorial virulence which has been related to thermotolerance, cell wall composition and maintenance, resistance to immune response, toxins, nutrient uptake during invasive growth, signaling regulation, and allergens. Resistance to azoles has also been reported and recognized as one of the important *A.fumigatus* pathogenesis-changing factors [15,16]. Also, many molecules or genes related to the pathogenicity of *A. fumigatus* have been found. Some of them are galactomannan glycoprotein encoded by afmp1, hydrophobic protein Rod A, fumagillin, gliotoxin, helvolic acid, fumigaclavin C and asp-hemolysin. These virulence factors are helpful for the pathogens surviving in the host and evading process of the immune system, such as masking the important PAMPs, inhibition of phagosome-lysosome fusion, production of antioxidants like catalase, SOD, and mannitol, or exerted multiple immunosuppressive actions on the host immunity by producing specific secondary metabolites such as gliotoxin (GT), fumagillin, actibind, and cytochalasin E [17]. *Aspergillus fumigatus* can grow at 55 °C and survive at temperatures above 70 °C. Five genes are associated with the thermotolerance of *A. fumigatus* (thtA, cgrA, afpmt1, kre2/afmnt1, and hsp1/aspf12). The essential gene for the growth of *A. fumigatus* over 37 °C is the afpmt1 gene which encodes for one mannosyl transferase [18]. *Aspergillus fumigatus* produce toxins which are mainly secondary metabolites of fungi and can affect the synthesis of DNA, RNA, and proteins, or alter the cell membrane and impair cellular functions. Some of them are diffusible toxic substances from conidia, mitogillin (res/mitF/aspf1), hemolysin (aspHS), gliotoxin (gliP and gliZ), verruculogen, fumagillin, and the transcription factor laeA. The most potent toxin produced by *A. fumigatus* is gliotoxin, whose immunosuppressive role relies on the impairment of macrophage phagocytosis, mitogen-activated T-cell proliferation, mast cell activation, cytotoxic T-cell response, monocyte apoptosis, and neutrophil function [19]. *A. fumigatus* can produce a large number of allergens; among them, 23 have their official names ranging from Asp f1 to Asp f34. Some of them show toxic or enzymatic activities, but some of them have been identified as adhesins [20,21]. It was suggested that the fungal cell wall has a mechanism similar to bacterial cell wall circulatory metabolism, which plays a role in the growth and reproduction of fungi [18]. The immune system is a very important part of *Aspergillus* spp. pathogenesis. Cellular immunity involves neutrophils, macrophages, dendritic cells and epithelial cells. The major components of humoral immune system are the complement system, antimicrobial peptides, collectins, acute phase proteins and circulating antibodies. Recognition by the complement system and activation of the cascade seems to interfere with fungal dissemination. *Aspergillus fumigatus* and *A. flavus*, the most virulent species, bind less C3 on their surface than non-pathogenic species [22]

However, recent studies have not yet identified the pathogenic factors which are unique to *Aspergillus* spp. This is one of the main reasons why it is critical for clinicians to find a unique diagnostic pattern when it comes to CPA.

## 3. Clinical Diagnosis

Early stages of CPA are usually clinically subtle, and a number of patients exhibit symptoms late in the course of the disease. The asymptomatic period can last 2–10 years, even longer in some cases. There are many clinical symptoms that can be found in patients with CPA, but none of them are specific only to this disease. Patients with a simple aspergilloma and *Aspergillus* nodules are typically asymptomatic. On the other hand, patients with chronic cavitary pulmonary aspergillosis (CCPA) and chronic fibrosing pulmonary aspergillosis (CFPA) will usually develop the clinical symptoms. The most common clinical symptoms of CPA are productive cough, hemoptysis, while systemic symptoms include weight loss and fatigue. Hou et al. showed that 92.8% of CPA patients presented with cough, 63.8% exhibited hemoptysis, while sputum production was present in 23.2% of patients [23]. Akram et al. in their retrospective study analyzed 218 CPA patients. The mean age of participants was 45.75 ± 6.26 years and 73.4% were males, and most subjects were non-smokers. It is well known that smoking is regarded as the major predisposing factor for development of COPD and other chronic lung conditions which are the base for CPA. Cough was the symptom in 95% of patients, fatigue in 92.7%, and sputum production in 90.4% of them. An amount of 85.8% of patients were with a fever, 59.6% had hemoptysis, weight loss in 34.9%, dyspnea in 17.9% and chest pain in 10.1% of patients [24]. On the other hand, Zhong et al., in a retrospective study, described clinical features in different subtypes of CPA and showed the presence of cough in 85% of CPA patients, expectoration in 70.7%, hemoptysis in 54.4%, and fever in 29.9% of subjects [25]. Weight loss occurs slowly during a longer period of time and may become very pronounced without treatment. Hemoptysis can vary from minor and occasional to extensive and life-threatening, causing significant blood loss [26]. As mentioned, patients can also experience fatigue, chest pain, and shortness of breath. Constitutional symptoms, such as fever and night sweats are unusual in these patients and if present they are usually related to the complication of CPA which leads to invasive fungal disease or another infection, such as bacterial pneumonia or TB [9]. Since none of these symptoms is specific to CPA, diagnosis cannot be based solely on the clinical picture. Chronic cough, weight loss, fatigue, night sweats and chest pain can also be attributed to PTB, which is the number one differential diagnosis for CPA. The study of Akrem et al. demonstrated previous lung TB in 44% of patients, and that active TB persisted in 18.8%. Other respiratory conditions included pulmonary sarcoidosis (21.1%), bronchiectasis 15.1%, asthma (10.6%), and COPD (10.6%) [24]. A study based in Pakistan found that TB was the underlying cause of CPA in 86.6% of patients followed by bronchiectasis caused by allergic bronchopulmonary aspergillosis (ABPA) in 11.9% [27]. On the other hand, a study conducted in the United Kingdom found that previous classical tuberculosis and non-tuberculous mycobacterial infection were the most common primary underlying conditions (15.3% and 14.9%, respectively). Others included allergic bronchopulmonary aspergillosis (ABPA), COPD and/or emphysema, pneumothorax and prior treated lung cancer [28]. Difficult to diagnose is the persistence of other lung diseases and conditions in CPA patients, due to masking and overlapping symptoms. Denning et al. showed that symptoms of underlying lung diseases can mask CPA comorbidity in a non-immunocompromised host. Emphysema and previous cavitary TB can mask CCPA, fibrosing CPA, aspergilloma, and nodule(s). Bronchiectasis can mask *Aspergillus*-bronchitis, whereas asthma can cover up ABPA [29]. COPD, a comorbidity that is often present in CPA patients, can mimic CPA symptoms, such as cough and shortness of breath, making it hard to distinguish the two diseases. Early-stage lung cancer may also present a diagnostic challenge as it can manifest with identical symptomatology and very similar radiological changes as CPA. A retrospective cohort study showed that CPA diagnosis is often missed in patients suspected of chest malignancy, which is considered to be the case due to low CPA awareness, as well as insufficiently specific diagnostic tools used in this patient population [30]. The most common extrapulmonaly chronic diseases in CPA patients are diabetes mellitus, autoimmune diseases and hypertension. 

## 4. Radiological Diagnosis

The radiological changes associated with CPA are the most significant proof for CPA diagnosis. Chest X-ray remains the most used imaging method worldwide, although CT provides a much more detailed visualization of the lung, and it is recommended for CPA diagnosis. Differential diagnosis of CPA includes lung cancer, metastases, cryptococcal nodules, coccidiomycosis and other pathogens [11]. When it comes to fungal conditions that can mimic CPA, such as coccidiomycosis, geographic location and travel history should be taken into consideration [11]. CPA is radiologically presented as multiple fungal balls in lungs, with cavitations and fibrosis [11,31,32]. One single fungal ball is called aspergilloma and it is usually formed in a pre-existing lung cavity, consisting mainly of *Aspergillus* hyphae and extracellular matrix. Other ubiquitous fungi could also form fungal balls in the lungs, but they are extremely rare, and the most common cause is *Aspergillus* [11]. Radiologically, CPA is presented usually as one or more cavities, typically with an irregular or thick wall, that tend to become larger over years, commonly forming pericavitary infiltrates and perforating into the pleura [11]. These cavities tend to affect the upper lobes [7] and they may or may not contain aspergilloma [31]. One of the previous studies showed that the most common imaging manifestations in CPA include cavitation (63.9%), fungal ball (36.7%), pleural thickening (32.0%), and bronchiectasis (31.3%) [25]. Although very sensitive and specific, a radiological diagnosis is not enough to make a conclusion that the patient suffers from CPA. CPA is presented by a combination of radiological findings and clinical symptoms present for at least 3 months [31]. 

It is very important to differentiate aspergilloma from serious lung conditions that require immediate specific therapy, such as cavitary lung cancer or bacterial lung abscess [33]. These diseases may often resemble aspergilloma on imaging, thus additional clinical information and sometimes lung biopsy are required for obtaining the definitive diagnosis. Lung tumor should always be included in differential diagnosis, as it can present in quite variable forms, one of those forms being a growth from a preexisting cystic mass, mimicking a fungus ball and making a diagnosis more challenging [34].

Chronic fibrosing pulmonary aspergillosis (CFPA) and chronic cavitary pulmonary aspergillosis (CCPA) are the most common complications of untreated CPA. CFPA is defined as irreversible fibrotic destruction of at least two lung lobes, leading to a progressive loss of the lung function [32]. Subacute invasive pulmonary aspergillosis (SAIA) is also the complication of CPA, and it shows many clinical and radiological similarities with CCPA, and commonly overlaps [31]. The main difference between SAIA and CCPA is a hyphal invasion of lung parenchyma that only happens in SAIA and can be detected if a biopsy is performed [35]. Furthermore, there is a difference in the time course of radiological progression; SAIA is characterized by more rapid progression that occurs over weeks rather than months [31]. SAIA usually affects immunocompromised individuals [31]. Patients with NTM infection may exhibit similar radiological changes as SAIA and CCPA patients, and therefore can present a diagnostic challenge [32]. It is often difficult to distinguish PTB from CPA, since PTB can also exhibit radiological features seen in CPA, such as cavitation, infiltrates, pleural thickening, and nodular formations. Furthermore, PTB also has the tendency to affect upper lung lobes. It is thought that the presence of intra-cavitary fungal ball, pleural thickening and paracavitary fibrosis are more commonly seen in patients with CPA, compared to patients with PTB [36]. A case report study by Higashi et al. showed that pulmonary actinomycosis can have an almost identical radiological presentation as pulmonary aspergilloma, as it presented on chest radiography and CT with multiple cavities, fibrosis and intracavitary nodular lesions [37]. Metastatic lesions present another possibility in differential diagnosis, as they can form cavities in the lung, resembling aspergilloma. So et al. reported a case of a patient with pulmonary metastasis of breast cancer presenting with a cavitary shadow and fungal ball-like masses on CT. They presumed that this pulmonary cavitation occurs through different mechanisms, including central degeneration and ischemic necrosis. They propose using bronchoscopy when evaluating patients with lung cavitary mimicking aspergilloma [38]. 

The implementation of fluorescence tomography to explore the complex lung microenvironments in the context of aspergillosis is promising. Lately, improvements to the specificity of radiographic imaging of invasive fungal infections have been attempted by coupling CT and positron emission tomography (PET) with [18F]fluorodeoxyglucose ([18F]FDG). [18F]FDG is a marker of metabolic activity well suited to cancer imaging, but with limited use in invasive fungal disease diagnostics due to its inability to differentiate between infectious etiologies, cancer, and inflammation [39,40]. Scientists used bioluminescence imaging using single genetically modified strains of *A. fumigatus*. It has enabled in vivo monitoring of IA in animal models of disease. Radiolabeled *Aspergillus*-specific monoclonal antibodies, and iron siderophores can be used for in vivo detection of *Aspergillus* lung infections in humans. Similar diagnostic procedures were not described for CPA, but there are some of experimental mice models which could refer to all forms of aspergillosis. Non-invasive imaging techniques of live infected mice include also combining bioluminescence but with magnetic resonance imaging (MRI) to both obtain dynamic information of fungal burden and lesion number and size in a non-invasive manner [41]. The late-stage disease is oxygen-limited, so inflammation can cause issues in luminescence resolution. In this case, MRI information is particularly beneficial. The hyphal-specific humanized monoclonal radiolabelled ([64Cu]DOTA-JF5) antibody hJF5, which has been used as a tool for the monitoring of pulmonary aspergillosis using antibody-guided positron emission tomography and magnetic resonance (immunoPET/MRI), has shown promise for clinical diagnostics purposes [42].

As stated above, none of the radiological findings in CPA are specific; hence, the additional microbiological and serological evidence of *Aspergillus* infection, as well as comparison with clinical symptoms, are needed in order to obtain the definitive diagnosis.

## 5. Laboratory Diagnosis of CPA

The laboratory abnormalities commonly seen in patients with CPA can vary depending on the individual and the stage of the disease. C-reactive protein (CRP) and erythrocyte sedimentation rate (ESR) may be elevated, indicating an ongoing inflammatory response [43]. Other inflammatory markers were also analyzed. Sehgal et al. hypothesized that plasma procalcitonin (PCT) may be elevated in CPA due to interferon-gamma (IFN-γ), which is decreased in CPA, down-regulating plasma PCT. In their study, which included 190 CPA cases and 40 controls, authors concluded that plasma procalcitonin performed poorly in diagnosing and monitoring treatment response in subjects with CPA [44]. Neutrophil-mediated inflammation is associated with disease activity [45]. Also, in Robinet et al.’s study, Th2 response is the main immune reaction, and the cytokines IL4, IL5, IL-15, TNF-α, and IL-10 were increased in peripheral blood [46]. Huang et al. have demonstrated that IL-6 and IL-1B serum levels were both significantly higher in the CPA group of patients than in the control group. Also in this study authors founded that systemic proinflammation was associated with disease severity and that PMN %, ESR, CRP, TNF-α, IL-6, IL-8 in peripheral blood increased with the severity of CPA in the univariate or multivariate analysis [47]. All of these pro-inflammatory cytokines could be potential biomarkers for CPA. Some of the proinflammatory cytokines could be useful to different CPA-TB from TB. Ren et al. showed significantly higher serum levels of numerous proinflammatory cytokines in peripheral blood of CPA-TB patients in comparison with TB patients. IL-8 levels alone provided the best discriminatory performance for distinguishing between TB and either CPA-TB patients or CPA patients [48]. Moreover, both IL-8 and TNF-α levels could be used to distinguish between TB and CPA-TB patients. Also, IL-8, TNF-α and IL-6 levels together could be used to distinguish between CPA-TB and TB patients. Zhong et al. showed a lower value of hemoglobin (HB) and serum albumin (ALB) with higher CRP and erythrocyte sedimentation rate in different form of CPA- SAIA and CFPA. SAIA, old age, male, low body mass index (BMI), COPD or emphysema, multiple distribution, low serum ALB, and positive sputum culture were adverse prognosis factors for SAIA and CCPA group, and BMI ≤ 20 kg/m^2^ was independently associated with increased mortality [25]. A higher-than-normal total white blood cell count, especially an increase in neutrophils, can be observed. In some cases, eosinophilia may be present [23,49]. These laboratory findings can provide supportive evidence for the diagnosis of CPA, but they are not definitive and conclusive for the final diagnosis. Microbiological and immunological evidence of *Aspergillus* infection is required for establishing CPA diagnosis, but only in addition to positive thoracic CT findings. Microscopy and culture of sputum and bronchial aspirate remain the reference standard but often lack sensitivity [50]. Direct microscopic examination in CPA can be helpful, as it is a well-established, cheap, and rapid test, and allows for the identification of fungal pathogens down to the genus level [26]. Isolation of *Aspergillus* cultures from respiratory samples is the basic method for diagnosing CPA, but it is not common to have microbiological confirmation by fungal isolation, even if CPA is suspected by radiological examination. While *A. fumigatus* is the number one causative agent of CPA [7], other species, most commonly *A. flavus* and *A. niger*, are also commonly found in bronchial aspirate of patients with CPA [51]. The main problem with the microbiological diagnosis of CPA is the ubiquitous nature of the fungus, so commonly positive culture means contamination from airborne spores or colonization of patient’s respiratory mucosa with fungi [11]. However, even colonization of respiratory mucosa should be carefully considered in immunocompromised individuals, as it could lead to the development of fungal disease of respiratory tract [52]. A study by Ohba et al. found that 67.4% of the patients have *Aspergillus* spp. colonization, and only 32.6% were diagnosed with CPA [51]. If the presence of *Aspergillus* sp. is isolated from bronchial aspirate and the patient has suspected CPA by radiological findings, and positive clinical symptoms of active respiratory tract disease, it could be considered as CPA [11]. Recent studies on microbiological testing do not overlook the appearance of azol-resistant A. fumigatus strains and their detection [15,16]. Singh et al. discovered resistance-associated mutations in 59% of CPA patients and 43% of ABPA patients. A G54 mutation, which confers itraconazole resistance, was found in 87.5% and 67% of patients with CPA and ABPA, respectively. Azole-resistant mutations were detected in 34% of BAL samples that were culture-negative but PCR-positive [16]. Because the detection of these azole-resistant strains could alter first-line therapy in patients with mutation [53], rapid techniques to detect resistance markers directly in respiratory samples are required. This is especially important in geographical areas where azole resistance is prevalent, with the recommendation to test all separate A. fumigatus colonies for azole resistance multiple times [16]. Serological diagnosis could be helpful in making decisions with CPA diagnosis, as elevated levels of *Aspergillus*-specific IgG antibodies are found in over 90% of patients with CPA, while *Aspergillus* precipitins are less sensitive [26]. In clinical practice, precipitation techniques have mostly been replaced by enzyme-linked immunosorbent assay (ELISA). *Aspergillus*-specific IgM antibodies have limited diagnostic value for CPA [54]. Although certain reports showed increased IgM antibodies in over 50% of CPA patients, this marker is considered to have low sensitivity and specificity. Regardless of the strong bactericidal and regulatory effects of IgM antibodies, its content in blood is low, its half-life is short, and it is susceptible to interference factors [55]. It can potentially have an early diagnostic value which *Aspergillus* IgG does not have, but more research is needed for further verification [56]. Detecting *Aspergillus*-specific IgG antibodies presents a key laboratory diagnostic tool in CPA and is the best noninvasive test for obtaining the diagnosis. A retrospective cohort study conducted in 2013 showed that 99% of CPA patients had positive serum *Aspergillus* precipitin IgG antibody test, while only 26% of the patients had positive sputum cultures [57]. On the other side, although the elevated level of IgG antibodies is highly sensitive to CPA, it lacks specificity, and it can also be present in other conditions, such as *Aspergillus* rhinosinusitis, *Aspergillus* bronchitis, allergic bronchopulmonary aspergillosis, etc. [43,58]. It is also important to mention that in CPA patients with hypogammaglobulinemia or other similar conditions *Aspergillus*-IgG can remain negative, due to that cause inability to produce an appropriate antibody response to an infection [31]. A study by Lee at al. found that more extensive forms of CPA, such as CCPA and CFPA, were related to higher *Asp*-IgG levels [59]. The main limitation of measuring the level of *Aspergillus*-IgG with the aim of diagnosing CPA is that *A. fumigatus* is not the only cause of CPA and there are no available serological tests for other species [60]. Value of *Aspergillus*-specific IgG levels is not important only for diagnosis, but also for follow-up CPA patients who are receiving antifungal therapy, as antibody titers fall slowly over time if treatment is successful [11], although there are opposite studies which report that the changes of *A. fumigatus*-specific IgG levels were not in correlation with treatment response [61]. The explanation for this could be the time needed for IgG antibody titers to fall, which could be individual and could depend on different clinical factors. Zhu et al. investigated the diagnostic laboratory findings in a retrospective CPA cohort. A total of 74 patients had positive Aspergillus IgG, the positivity rate was 72.1% in CCPA, 75.0% in chronic necrotizing pulmonary aspergillosis (CNPA), 41.7% in SA, and 30.3% in *Aspergillus* nodes (AN) patients. The CCPA and CNPA patients exhibited significantly higher median levels of *Aspergillus* IgG antibody than the SA and AN patients, while antibody levels were similar between *A. fumigatus* and non-*A. fumigatus* cases. *Aspergillus* IgG was negative in a total of 52 CPA patients, who were diagnosed by histopathology or sputum fungal culture. This study also evaluated the performance of a novel Aspergillus IgG lateral flow assay (LFA) which was highly sensitive for *A. fumigatus*-associated CCPA (96.2%). This novel LFA has a satisfactory performance and allows earlier screening and diagnosis of CPA patients [61].

β-1,3-D-glucan (βDG) is not specific, while galactomannan, a cell wall component of *Aspergillus* sp., is specific but not sensitive, so these markers for respiratory samples are not useful for the diagnosis of CPA [62,63,64,65]. Falcao de Oliveira demonstrated that bronchoalveolar lavage galactomannan (73%), serology by immunodiffusion test (81%), and histology (78%) had the best sensitivity in CPA patients. They analyzed the counterimmunoelectrophoresis (CIE) titers and CRP in different subtypes of CPA. CIE was increased in CFPA and SAIA, whereas CRP generally presented lower values, with higher values in SAIA and lower values for the Aspergillus nodule. Authors concluded that the inflammatory markers and CIE titers tend to be higher in forms of the more extensive lung parenchyma involvement, such as SAIA and CFPA [66].

## 6. Conclusions

CPA can be challenging to diagnose due to its non-specific symptoms and similarities with other respiratory conditions combined with poor awareness of the medical community about the disease. This can result in delayed treatment, even for years, and the worsening of the patients’ condition. Serological tests certainly play a significant role in diagnosing CPA but cannot be interpreted without radiological confirmation of CPA. Although many data are published on this hot topic, there is still no single definitive test for diagnosing CPA, and a multidisciplinary approach, which involves a combination of the clinical picture, radiological findings, microbiological results and exclusion of other mimicking diseases, is essential for the accurate diagnosis of CPA. The most promising for diagnosis and therapy monitoring are molecular CT/NMR/PET methods, such as hyphal-specific humanized monoclonal radiolabeled antibodies which could be used for in vivo detection of *Aspergillus* lung infections in humans.

## Data Availability

Data sharing not applicable.
